# Exploring multidimensional operationalizations of precarious employment in Swedish register data – a typological approach and a summative score approach

**DOI:** 10.5271/sjweh.3928

**Published:** 2021-03-01

**Authors:** Johanna Jonsson, Nuria Matilla-Santander, Bertina Kreshpaj, Cecilia Orellana, Gun Johansson, Bo Burström, Magnus Alderling, Trevor Peckham, Katarina Kjellberg, Jenny Selander, Per-Olof Östergren, Theo Bodin

**Affiliations:** Unit of Occupational Medicine, Institute of Environmental Medicine, Karolinska Institutet, Stockholm, Sweden; Center for Occupational and Environmental Medicine, Stockholm Region, Stockholm, Sweden; Equity and Health Policy Research Group, Department of Global Public Health, Karolinska Institutet, Stockholm, Sweden; Centre for Epidemiology and Community Medicine, Stockholm Region, Stockholm, Sweden; Department of Environmental and Occupational Health Sciences, University of Washington, Seattle, USA; Social Medicine and Global Health, Department of Clinical Sciences Malmö, Lund University, Malmö, Sweden

**Keywords:** employment condition, nonstandard employment, employment quality, epidemiology, occupational health

## Abstract

**Objectives:**

This study aimed to explore multidimensional operationalizations of precarious employment (PE) in Swedish register data using two approaches: (i) a typological approach and (ii) a dimensional, summative scale approach. It also examined the distribution of sociodemographic and occupational characteristics of precarious employees in Sweden.

**Method:**

Register data was retrieved on individuals and their employers in the Swedish workforce. Five items corresponding to three dimensions of PE were operationalized: contractual relationship insecurity, contractual temporariness, multiple jobs/sectors, income level, and lack of unionization. First, latent class analysis was applied and a typology of six employment types emerged. Second, a summative scale was constructed by scoring all PE-items.

**Results:**

Three types of PE were found using the typological approach, which were characterized by direct employment, solo self-employment and multiple job holding, respectively. The summative scale score ranged between -10 and +2 (average: -1.8). Particularly poor scores were seen for solo self-employed, multiple job holders/multiple sectors, and low income. Female gender, young age, low education and foreign origin were prone to precariousness. PE was more frequent among certain economic sectors and occupations.

**Conclusions:**

Using an existing register of labor market data, two operationalizations of PE were constructed and rendered promising for exposure assessment. Hence, the operationalizations could be of interest for countries with similar data structure. Both approaches highlighted precarious combinations of employment conditions and pointed towards the existence of a wide continuum of precariousness on the labor market. Etiological studies and research assessing trends over time are needed to validate these findings.

Precarious employment (PE) is recognized as a multi-dimensional construct encompassing several aspects of employment conditions, including lack of protective regulation, short/uncertain employment duration, lack of fringe benefits and poor wages (1–6). Yet, no internationally accepted definition transcending historical and socio-political contexts currently exists (5, 6). In addition, unidimensional measures such as type of employment [eg, (7–9)] are still widely applied to operationalize PE in epidemiological research. However, unidimensional indicators do not fully capture the extent of precariousness, limiting our ability to monitor the prevalence, distribution and health effects of PE (10). In order to move towards a more comprehensive operationalization, several efforts have been dedicated to developing multidimensional PE measures. This has been done using both typological measurement approaches, where employment sharing certain features are grouped together (11–14), and dimensional approaches, through scales (4, 15) and indices (16), in which PE is represented on a continuum from low to high.

Despite significant interest in this area, several limitations are present in prior studies using multidimensional PE measures. For one, the self-employed are often not included, despite the fact that these workers lack many of the rights and protections of regular employees. Solo self-employed can be considered particularly vulnerable in regards of income and job insecurity, as well as in terms of economic pressures and downturns (17, 18). Further, many previous operationalizations have relied primarily on survey data, such as Europe-wide surveys like the European Working Conditions Survey (EWCS) (11, 12, 14) and the European Labor Force Survey (EU-LFS) (18), as well as country-specific surveys from, for example, Canada (16, 19), the US (13), Spain (4, 20), and Sweden (15, 21). Drawing conclusions from survey samples involves risk of bias. Under- and over-coverage in sampling frames, high proxy rates (22), and non-response rates (22, 23) have been reported for the EU-LFS (22) and the EWCS (23). Also, there have been reports of decreasing response rates and other sources of bias in national surveys within Sweden (24–26).

Alternatively, Sweden and other Nordic countries have comprehensive register structures containing several linkable population-based registers with detailed data on employees (including the self-employed) and employers. Register-based operationalizations of PE would circumvent some of the challenges of survey-based research and could also provide a more precise picture of the sociodemographic and occupational characteristics over-represented in PE conditions. Further, studies that researchers struggle with could be enabled, such as surveillance of the PE population and longitudinal studies of various social and health effects of PE, particularly if both a typological and dimensional approach could be explored and applied in epidemiological research.

## Objectives

The objective of this study was to explore multidimensional operationalizations of PE in Swedish register data using two approaches (i) a typological approach and (ii) a dimensional, summative scale approach. We also aimed to examine the distribution of sociodemographic and occupational characteristics of precarious employees in Sweden.

## Method

### Data and data collection

Register data was collected from the Longitudinal Integrated Database for Health Insurance and Labour Market Studies (LISA) for the year 2014. LISA is held by Statistics Sweden and covers the population of Sweden from the age of 15 onwards; it is updated annually and includes both individual- and employer-level data (27).

Individual-level data was retrieved on age (18–24; 25–34; 35–44; 45–54; 55–65 years), gender (female; male), highest completed education (primary school; secondary school; tertiary education ≤2 years; tertiary education ≥3 years), country of birth (Sweden; born in a Nordic country; born in EU-28; born outside EU-28), occupation, income (annual salary from employer; annual income from other work-related sources), income from unemployment insurance and study compensation (yes; no). Data was further collected on individuals’ employers, including reference employer (largest source of income in November) and primary, secondary and tertiary employers (largest to third largest source of income during the year), economic sector (grouped in 10 and 15 categories), number of employees in the company (1; 2–5; 6–10; 11–50; 51–100; >100) and ownership sector (private; public). Reference employer was also retrieved for year 2012 and 2013. Individuals were linked across years with the use of an (anonymized) identification number replacing the unique Swedish personal identification number.

### Study population

In 2014, LISA included 6 728 752 individuals. Individuals were included in the study if they were alive and residing in Sweden at the end of the year, had at least one employer, a registered work-related income, and were 18–65 years of age. Individuals with missing values in any of the items required for the PE operationalizations were excluded. The final study population was 4 349 322 (supplementary material www.sjweh.fi/show_abstract.php?abstract_id=3928, figure S1).

### Procedure

*Conceptual framework of PE*. Operationalization of PE was based on a review by Kreshpaj et al (6) who identified three dimensions and nine themes of PE: (i) employment insecurity, including items of contractual relationship insecurity (contract with employer or with other party, eg, agency or self-employed), contractual temporariness (permanent or fixed-term contract), underemployment (full-time or part-time contract) and multiple jobs and/or multiple jobs in multiple economic sectors; (ii) income inadequacy, including income level (low hourly wage, monthly income or annual income); and (iii) lack of rights and protection, including lack of unionization (representation at the work place), lack of social security (social support/benefits), lack of regulatory support (labor policies) and lack of work place rights (actual and/or power to exercise work place rights).

*Operationalization of PE*. A total of five items representing all three dimensions were found to be operationalizable (table 1). Income level was operationalized in two steps. First, the total estimated annual salary (before taxes) was estimated by summing up work-related income sources, ie, salary, income from business, work-related social insurance benefits (parental benefits, sickness benefits and related sources) and unemployment benefits. In order to estimate the full annual salary (100%), the social insurance and unemployment benefits were multiplied by 1.25 as these are paid out in approximately 80% of the monthly salary (28, 29). This estimation was done so that the emerging income level was not affected by temporary absences, such as parental leave, sickness absence or unemployment. Second, the estimated annual salary was categorized based on the median salary of the population meeting the inclusion criteria of 2014 (325 400 Swedish krona): <60%, 60–79%, 80–119%, 120–199% and ≥200% of the median. The <60% cut-off was chosen in order to account for individuals living at risk of poverty (30).

**Table 1:** Operationalization of items of precarious employment with the use of register data. [SNI=Swedish Standard Industrial Classification.]

Dimension	Theme	Item specification	Operationalization
Employment Insecurity	Contractual relationship insecurity	(1) Directly employed by the employer (2) Employed by an agency (3) Combination of self-and direct employment (4) Self-employed (5) Solo self-employed	(1) Employed directly by employer, while not being identified by (2), (3), (4) or (5) (2) Employed directly by employer and employers’ workplace activity is “Temporary employment agency activities” (SNI-code = 78.2) (3) Employed directly by employer and self-employed (4) Self-employed or self-employed in corporation, where number of employees is >1 (5) Self-employed or self-employed in corporation, where number of employees is =1
	Contractual temporariness	(1) Stable employment (2) Unstable employment	(1) Having the same employer for 3 years ^a^ (2) Having the same employer for <3 years
	Underemployment Multiple jobs/ economic sectors	Full-time vs. part-time employment (1) Having one job (employer) during the current year (2) Having multiple jobs (3) Having multiple jobs in multiple sectors	No suitable operationalization identified (1) 1 job (2) ≥2 jobs ^b^ (3) ≥2 jobs in >1 economic sector ^c^
Income Inadequacy	Income level	Income level (before taxes) in relation to the median of the population	(1) ≥200% of the median ^d^ (2) 120–199% of the median ^e^ (3) 80–119% of the median ^f^ (4) 60–79% of the median ^g^ (5) <60% of the median ^h^
Lack of rights and protection	Lack of unionization	Likelihood of being covered by collective bargaining agreement in the company of employment	(1) >90% (2) 71–90% (3) ≤70%
	Lack of social protection Lack of regulatory support Lack of workplace rights	Social protection/ benefits/ household income Labour policies/standards Workplace rights	No suitable operationalization identified No suitable operationalization identified No suitable operationalization identified

a Operationalized by assessing reference employer for year 2012 and 2013, in addition to 2014.

b Operationalized by adding up the number of unique employers during the year, ie, the reference employer, primary, secondary and tertiary employer.

c Agriculture, commerce and hospitality, construction, education, financial services, health, industry, other services, public administration, transport.

d >650 800 Swedish krona.

e 390 480–650 800 Swedish krona.

f 260 320–390 480 Swedish krona.

g 195 240–260 320 Swedish krona.

h 100–195 240 Swedish krona.

Further, union coverage was operationalized as the approximate likelihood of being covered by a collective bargaining agreement (CBA) at the company level. Likelihood of coverage was calculated as the probability of certain groups being covered by occupational pension, using data from the Swedish Social Insurance Inspectorate (31). CBA coverage was estimated by multiplying the probabilities reported for company size, ownership sector and economic sector (15 categories), stratified by gender. Public sector employees were considered 100% covered by CBA, and solo self-employed were considered 0% covered. See details in supplementary table S1.

*Constructing an employment typology*. Latent class analysis (LCA) was applied in order to extract clusters – employment types – from the data. First, the LCA was run on an exploratory dataset containing half of the sample. The initially best cluster solutions were chosen based on plotting Akaike Information Criterion (AIC) and Bayesian Information Criterion (BIC). Thereafter, test statistics for relative fit and measures of classification diagnostics were compared. The former included AIC, BIC, and sample-size adjusted BIC (SABIC). The latter included entropy and average posterior probabilities. Furthermore, conditional item probabilities (ie, the likelihood of endorsing items given a specific class membership) and latent class homogeneity and separation (observed versus expected probability ratio) were inspected in order to find the most informative solution. Second, a cross-validation was conducted on the calibration data set (ie, the other half of the sample). Finally, the chosen cluster solution was run for the full dataset. See supplementary table S2 for details on the exploratory and confirmatory solutions.

A six-cluster solution was chosen as the best fit. According to AIC, BIC and SABIC, a seven-cluster solution was the best, while the entropy and average posterior probabilities were slightly better for four- and five-cluster solutions. However, importantly, when comparing the unique high conditional item probabilities, a six-cluster solution resulted in more distinct clusters. Conditional item probabilities for the six-cluster solution are shown in table S3. Labels were assigned to each cluster by inspecting conditional item probabilities and confirmed by assessing the distribution of sociodemographic characteristics across employment types.

*Constructing a summative scale*. Levels of PE items were scored based on their relative deviation from the “standard” level on an ordinal scale (where applicable). Standard levels – ie, direct employment, stable employment, one job, median salary and >90% CBA coverage – were scored as 0, while lower and higher scores were given for deviations from the standard levels (-2 to +2). See scoring of PE items in table 2.

**Table 2:** Scoring of items of precarious employment.

Item	Score				

	-2	-1	0	1	2
Contractual relationship insecurity	Solo self-employed	Self- and direct employment	Directly employed		
		Self-employed			
		Agency employed			
Contractual temporariness	Unstable employment		Stable employment		
Multiple jobs/ economic sectors	≥2 jobs in >1 sector	≥2 jobs	1 job		
Income(% of median)	<60	60–79	80–119	120–199	≥200
Collective bargaining agreement coverage (% likelihood)	≤70	71–90	91–100		

For low earners, holding multiple jobs and frequently changing employer could reflect a weak position on the labor market, whereas the reverse could be true for high earners whose skills are in high demand. For these workers, such as successful freelancers/consultants, multiple jobs could allow for skill acquisition, receiving additional credentials or moving into a new occupation (32), off-setting detrimental impacts of short job tenure or being self-employed. Hence, to reduce misclassification, positive scores were introduced for those in the highest income categories. All items received the same weight, consistent with previous studies constructing scales of PE (11, 16).

### Further statistical analysis

LCA modelling was conducted for the sample excluding students in order to detect the potential effects of this group. Descriptive tables and figures were created for the total population and stratified per employment type (using modal assignment, ie, most likely cluster). In order to compare the resulting typology and summative scale, both measures were described in terms of characteristics considered especially relevant for PE, including gender, age, level of education, country of birth, occupation (where applicable) and economic sector (2, 11, 14). Finally, the proportion of each employment type falling below the 25^th^ percentile of the summative score for the total population was calculated to identify the most precarious population. LCA modelling was conducted in Mplus version 8.4 (33), and data management and descriptive statistics were performed with SAS, version 9.4 (SAS Institute, Cary, NC, USA).

### Ethical considerations

The Regional Ethics Committee of Stockholm approved this study (2016/2325-31).

## Results

### Employment typology

Of the six emerging employment types, three were considered non-precarious and three were considered precarious, as reflected by their labels. The non-precarious employment types were labelled (i) “standard employment relationship” (SER-type; 60%), characterized by large proportions of direct and stable employment, one employer, median income and high CBA coverage; (ii) “business owners” (2%) that were non-solo self-employed with one job, stable employment, median-to-high income and moderate CBA coverage; and (iii) “proficians” (10%) mainly in direct employment, either stable or unstable, in multiple jobs/multiple sectors, with high income. The three precarious employment types were labelled (i) “PE relationship” (PER-type; 22%), characterized by large proportions of direct- and agency-employment, unstable employment, multiple jobs/multiple sectors, and poor income; (ii) “precarious self-employment” (5%) with large proportions of solo self-employment, one job, poor income and low CBA coverage; and (iii) “precarious multiple job holders” (2%), being in combined employment (employment and self-employment), with multiple jobs in multiple sectors, poor-to-median income and low CBA coverage. Further rationale for the labels is provided in table S4. Descriptive statistics for the PE items by employment type are presented in table 3. Excluding students did not affect the interpretation of the typology (data not shown).

**Table 3:** Distribution of items of precarious employment (scoring within brackets) for the typology ^a, b^ and the total population with average summative scale scores and standard deviations (SD). [SER=standard employment relationship; BO=business owners; PER=precarious employment relationship; P-SE=precarious self-employment; P-MJH=precarious multiple job holders; CBA=collective bargaining agreement.]

	SER		BO		Proficians		PER		P-SE		P-MJH		Total			
						
	N	%	N	%	N	%	N	%	N	%	N	%	N	%	Score	SD
Total	2 593 238	60	89 511	2	422 933	10	947 882	22	199 630	5	96 128	2	4 349 322	100	-1.8	2.4
Contractual relationship insecurity																
Directly employed by the employer (0)	2 409 239	91	0	0	300 122	71	859 475	91	30 294	15	0	0	3 599 130	83	-1.4	2.2
Employed by an agency (-1)	11 291	0	0	0	1 064	0	42 674	5	0	0	0	0	55 029	1	-4.2	2.2
Combination of self- and direct employment (-1)	172 708	7	0	0	121 378	29	42 753	5	6 622	3	90 991	95	434 452	10	-3.2	2.5
Self-employed (-1)	0	0	89 511	100	369	0	2 980	0	28 455	14	3 971	4	125 286	3	-2.8	1.9
Solo self-employed (-2)	0	0	0	0	0	0	0	0	134 259	67	1 166	1	135 425	3	-5.3	1.6
Contractual temporariness																
Stable employment (0)	2 337 085	90	85 465	95	257 716	61	31 247	3	157 920	79	42 209	44	2 911 642	67	-0.7	1.7
Unstable employment (-2)	256 153	10	4 046	5	165 217	39	916 653	97	41 710	21	53 919	56	1 437 680	33	-4.1	1.9
Multiple jobs/economic sectors																
1 job (0)	2 462 364	95	83 852	94	257	0	364 989	39	193 124	97	705	1	3 105 291	71	-1.0	2.0
≥2 jobs (-1)	0	0	3 670	4	172 476	41	211 211	22	5 345	3	30 456	32	423 158	10	-3.3	2.0
≥2 jobs in >1 economic sector (-2)	130 874	5	1 989	2	250 200	59	371 682	39	1 161	1	64 967	68	820 873	19	-4.1	2.1
Income level (% of median)																
≥200 (2)	147 649	6	6 635	7	61 572	15	2 099	0	2 167	1	3 297	3	223 419	5	0.6	1.6
120–199 (1)	718 921	28	45 409	51	265 919	63	13 502	1	29 230	15	20 073	21	1 093 054	25	-0.4	1.7
80–119 (0)	353 337	52	33 102	37	84 590	20	254 833	27	35 459	18	27 251	28	1 788 572	41	-1.4	1.7
60–79 (-1)	261 192	10	2 610	3	10 852	3	220 159	23	47 334	24	16 464	17	558 611	13	-3.0	1.9
<60 (-2)	112 139	4	1 755	2	0	0	457 289	48	85 440	43	29 043	30	685 666	16	-5.1	1.7
CBA coverage (% likelihood)																
>90 (0)	255 747	87	16 292	18	350 843	83	582 253	61	0	0	3 316	3	3 208 451	74	-1.1	2.0
71–90 (-1)	277 117	11	32 580	36	64 953	15	233 301	25	0	0	9 623	10	617 574	14	-3.0	2.1
≤70 (-2)	60 374	2	40 639	45	7 137	2	132 328	14	199 630	100	83 189	87	523 297	12	-4.8	2.0

a Modal assignment.

b Employment types are ordered from highest to lowest average summative scale scores.

### Summative scale

**Figure 1:**
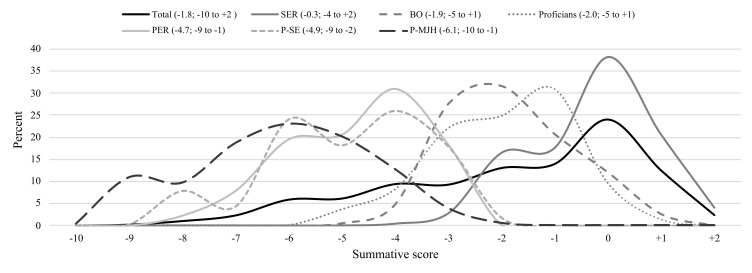
Distribution of summative scale scores for the typology and the total population (average and min to max summative scores within brackets). [SER=standard employment relationship; BO=business owners; PER=precarious employment relationship; P-SE=precarious self-employment; P-MJH=precarious multiple job holders.]

The overall summative score ranged between -10 and +2 with an average of -1.8. In particular, agency employment (-4.2), solo self-employment (-5.3), unstable employment (-4.1), multiple jobs in multiple sectors (-4.1), income <60% of the median (-5.1) and CBA coverage ≤70% (-4.8) were associated with poor scores (see table 3). Approximately 25% of the total population scored 0. The SER-type had the highest score with an average of -0.3, while the business owners and proficians had scores of -1.9 and -2.0, respectively. The PER-type, precarious self-employed and precarious multiple job holders had the lowest scores with averages of -4.7, -4.9, and -6.1, respectively. See the score distribution in figure 1.

The 25^th^ percentile of the summative score for the total population was -4. Only 0.4% of the SER type, and 5.3% and 11.8% of the business owners and proficians, respectively, had scores below -4. Meanwhile 81.5%, 80.4% and 95.7% of the PER-type, precarious self-employed and precarious multiple job holders, respectively, were captured by the lowest quartile (data not shown).

### Sociodemographic and occupational characteristics

Sociodemographic characteristics are presented in table 4. Compared with the SER-type, the PER-type had a greater proportion of women (53% versus 50%), 18–24 year-olds (34% versus 4%) and individuals working within sectors of accommodation and food services (9% versus 2%) and professional, scientific and technical activities (16% versus 9%). Additionally, the PER-type had comparatively less individuals with tertiary education >3 years (18% versus 28%) and individuals born in Sweden (79% versus 86%). The most common occupational groups of the PER-type included food preparation assistants (49%), agriculture laborers (46%) and sales and services workers (35%), in contrast with occupations of armed forces (85–86%) and banking, financial and insurance managers (84%) for the SER-type. See Figure S2a-b. The average summative scale scores within occupations were substantially lower for the PER-type, compared to the SER-type, for every occupation displayed.

**Table 4:** Sociodemographic characteristics for the typology ^a, b^ and the total with average summative scale scores and standard deviations (SD). [SER=standard employment relationship; BO=business owners; PER=precarious employment relationship; P-SE=precarious self-employment; PMJH=precarious multiple job holders]

	SER		BO		Proficians		PER		P-SE		P-MJH		Total			
						
	N	%	N	%	N	%	N	%	N	%	N	%	N	%	Score	SD
Gender																
Male	1 286 373	50	72 151	81	248 836	59	442 828	47	133 142	67	59 283	62	2 242 613	52	-1.7	2.4
Female	1 306 865	50	17 360	19	174 097	41	505 054	53	66 488	33	36 845	38	2 106 709	48	-1.9	2.3
Age (years)																
18–24	110 196	4	440	0	9 035	2	326 818	34	6 673	3	4 849	5	458 011	11	-4.2	2.1
25–34	511 817	20	8 790	10	74 707	18	293 429	31	28 810	14	18 964	20	936 517	22	-2.3	2.3
35–44	675 115	26	25 300	28	122 416	29	152 464	16	46 422	23	24 167	25	1 045 884	24	-1.4	2.3
45–54	718 371	28	32 726	37	130 513	31	110 408	12	60 184	30	26 496	28	1 078 698	25	-1.2	2.2
55–65	577 739	22	22 255	25	86 262	20	64 763	7	57 541	29	21 652	23	830 212	19	-1.2	2.2
Education																
Primary	225 179	9	13 208	15	24 273	6	114 941	12	33 229	17	9 640	10	420 470	10	-2.4	2.4
Secondary	1 251 967	48	47 686	53	160 157	38	503 053	53	104 220	52	45 261	47	2 112 344	49	-2.0	2.3
Tertiary <2 years	387 794	15	12 042	13	68 331	16	143 491	15	27 782	14	17 895	19	657 335	15	-1.8	2.5
Tertiary >3 years	716 336	28	16 182	18	169 039	40	172 099	18	31 955	16	22 883	24	1 128 494	26	-1.2	2.2
Missing	11 962	0	393	0	113	0	14 298	2	2 444	1	449	0	30 679	1		
Country of birth																
Sweden	2 230 106	86	78 097	87	364 849	86	746 154	79	158 682	79	81 755	85	3 659 643	84	-1.7	2.4
Nordic countries	57 942	2	1 657	2	8 267	2	13 480	1	4 495	2	1 654	2	87 495	2	-1.4	2.2
EU-28	73 008	3	2 460	3	11 241	3	38 846	4	10 379	5	3 478	4	139 412	3	-2.3	2.5
Outside EU-28	214 524	8	6 538	7	35 491	8	143 007	15	24 212	12	8 418	9	432 190	10	-2.4	2.4
Unknown	82	0	5	0	18	0	107	0	9	0	4	0	225	0	-3.0	2.3
Missing	17 576	1	754	1	3 067	1	6 288	1	1 853	1	819	1	30 357	1		
Studied during year																
No	2 553 347	98	89 387	100	420 437	99	728 305	77	195 815	98	91 155	95	4 078 446	94	-1.6	2.3
Yes	39 891	2	124	0	2 496	1	219 577	23	3 815	2	4 973	2	270 876	6	-4.8	1.8
Ownership sector																
Private	1 541 205	59	89 439	100	266 927	63	719 722	76	199 630	100	94 355	98	2 911 278	67	-2.2	2.5
Public	1 052 033	41	72	0	156 006	37	228 160	24	0	0	1 773	2	1 438 044	33	-1.1	1.8
Economic sector																
Electricity, Gas, Steam and Air Conditioning Supply; Water Supply; Sewerage, Waste Management and Remediation Activities	35 914	1	245	0	5 945	1	4 588	0	175	0	132	0	46 999	1	-0.6	1.9
Agriculture, Forestry and Fishing	13 143	1	2 707	3	2 224	1	10 889	1	19 442	10	8 728	9	57 133	1	-4.3	2.1
Mining and Quarrying; Manufacturing	416 080	16	10 313	12	51 736	12	57 100	6	8 184	4	3 194	3	546 607	13	-0.7	1.9
Construction	161 769	6	17 605	20	26 455	6	59 466	6	30 096	15	10 134	11	305 525	7	-2.0	2.4
Wholesale and Retail Trade	309 359	12	15 963	18	39 561	9	138 695	15	28 349	14	10 360	11	542 287	12	-2.0	2.4
Transportation and Storage	128 650	5	4 951	6	20 854	5	51 404	5	8 531	4	4 526	5	218 916	5	-1.9	2.2
Accommodation and Food Service Activities	40 108	2	5 227	6	6 132	1	84 775	9	12 674	6	5 351	6	154 267	4	-4.3	2.1
Information and Communication	102 476	4	5 508	6	24 778	6	23 017	2	10 476	5	5 517	6	171 772	4	-1.2	2.4
Financial and Insurance Activities	64 397	2	529	1	11 794	3	11 052	1	918	0	832	1	89 522	2	-0.6	2.1
Real Estate Activities	35 528	1	1 195	1	7 011	2	14 956	2	3 733	2	2 615	3	65 038	2	-2.3	2.5
Professional, Scientific and Technical Activities; Administrative and Support Service Activities	223 626	9	17 153	19	46 747	11	153 684	16	37 712	19	21 229	22	500 151	12	-2.6	2.5
Public Administration and Defence	195 706	8	9	0	35 156	8	28 411	3	12	0	201	0	259 495	6	-0.7	1.8
Education	319 848	12	1 360	2	46 383	11	94 717	10	32 58	2	4 146	4	469 712	11	-1.6	2.0
Human Health and Social Work Activities	477 936	18	3 951	4	79 016	19	160 645	17	6 917	3	4 722	5	733 187	17	-1.5	2.0
Arts, Entertainment and Recreation; Other service activities	68 698	3	2 795	3	19 141	5	54 483	6	29 153	15	14 441	15	188 711	4	-3.5	2.5

a Modal assignment.

b Employment types are ordered from highest to lowest average summative scale scores.

The precarious self-employment type was characterized by high proportions of males (67% versus 50% in the SER-type), 55–64 year-olds (29% versus 22%) and individuals with elementary education (17% versus 9%), as well as comparatively more individuals from the arts, entertainment and recreation (15% versus 3%) and construction sectors (15% versus 6%). This employment type also had less individuals born in Sweden (79% versus 86%).

The precarious multiple job holders had a large proportion of males (62%, compared with 50% of the SER-type), while age was similarly distributed among the employment types. In addition, there was a slightly larger proportion of ≤2 year tertiary education (19% versus 15%), and a slightly smaller proportion of ≥3 year tertiary education (24% versus 28%). Work was mainly carried out in the private ownership sector (98% versus 59%), within sectors of professional, scientific and technical activities (22% versus 9%) and agriculture (9% versus 1%).

In accordance with the precarious employment types, the lowest average summative scores were found among women (-1.9), 18–24 year-olds (-4.2), individuals with primary school education (-2.4), foreign-born outside the EU-28 (-2.4), students (-4.8), private ownership sector workers (-2.2), as well as within certain economic sectors, especially agriculture (-4.3), accommodation and food service (-4.3), and arts and entertainment (-3.5).

## Discussion

### Key results

*Typological approach*. In summary, we found three types of PE using a typological measurement approach: one in employment (22%) (PER), one in solo self-employment (5%), and one holding multiple jobs in combination-employment (2%). The size of the PER-type (as well as all precarious types taken together) and the SER-type were in the range of other estimates (11, 34, 35).

The employment types characterized by self-employment and multiple job holding represent a novel aspect of this study. A study by Peckham et al (13) conducted in the US and a study by Gevaert et al (14) using data from the EWCS applied the construct of employment quality and identified two and four types of self-employed, respectively, in their typological measurement approaches. In both the US and European analyses, insecure self-employed types emerged, although these are not entirely comparable to types of precarious self-employment and multiple job holders reported here. This is in part because the employment quality concept extends the concept of PE as well as the fact that neither the US nor European analyses accounted for combined employment. Both our study and the European one, however, indicate that the number of employees of self-employed is an important indicator in distinguishing between different forms of self-employment. Our study also suggests that combined employment is a useful indicator.

*Summative scale approach*. The average of the summative scale score was -1.8, with approximately 25% of the total population scoring 0. In this approach, 0 represents standard employment conditions in all dimensions with the possibility of receiving 1–2 additional points for incomes >120% of the median. The negative overall average score was therefore expected. Others have developed PE scales, the most notable being the Employment Precariousness Scale (4) and the Employment Precarity Index (16). The index identified precarious conditions by the upper quartile, while studies applying the Employment Precariousness Scale successfully implemented the use of tertiles, quartiles and quintiles in relation to health outcomes and social consequences (36–38). Suitable cut-offs to determine PE for the present scale will have to be determined in future studies, but the lowest quartile captures the majority of the precarious employment types identified in our data.

### Sociodemographic and occupational characteristics of precarious employees

Previous studies have indicated that workers with PE arrangements are predominantly female and young (2, 17, 37). In the current study, women had a slightly poorer average summative score than men and were in slight majority in the PER-type. Young individuals were more clearly overrepresented in the PER-type and by low scores. By including the self-employed in our analysis, we provide a nuanced picture of PE in the Swedish context. Importantly, we show that many men and older age groups experience PE, but that the character of precariousness might differ between genders and age. Women and young might be overrepresented in terms of more “traditional” precariousness, ie, more often characterized by direct, but unstable, employment in certain economic sectors and occupations. Meanwhile men and older ages might be overrepresented in precarious self-employment and precarious multiple job holding. The previous has been supported by Gevaert et al (14).

As expected, foreign-born individuals showed lower summative scores as compared to native Swedes, and were overrepresented in the PER and precarious self-employment types. The precariousness of foreign-born, recent immigrants and racialized workers has been reported by others (17), pointing towards an ethnicization of the precarious work force. In Sweden, foreign-born are more often self-employed, which reportedly is due to the lack of employment options, while Swedish-born more often combine employment with self-employment (34). The latter is evident when comparing the proportion of Swedish-born among precarious multiple job holders and precarious self-employed. Finally, low education was found to be prevalent among the precarious, especially for the PER and precarious self-employment types, in accordance with previous reports (2, 14, 17).

Economic sectors and occupations with low summative scale scores found in this study is partly confirmed by a Eurofund report constructing employment types and scores of employment quality (11). The report found low scores for agriculture grouped together with mining and quarrying (although the latter received high scores in this study), transportation and storage, and wholesale and retail. In our study, elementary occupations (eg, food preparation assistants), skilled agricultural workers, and service and sales workers received low scores. These occupations were also prevalent within the precarious employment types identified by Eurofund, as well as other studies using Canadian data (17).

### Consistency of employment types and summative scale score

Cross-checking the summative scores and the employment types by comparing the proportion of each type falling under the lowest quartile of the total score, confirms that the lowest quartile captures the majority of all precarious types. Further, sociodemographic groups known to be associated with employment precariousness were overrepresented in the precarious employment types – especially the PER-type – and generally received lower scores. This indicates that the employment types and the scores are consistent and that both capture PE.

### Strengths and weaknesses of the two approaches

Both methods find strength in that they are based on a solid theoretical and empirical foundation and thus easily interpreted, despite the underlying multidimensionality. The typological approach has an additional strength in that it provides a nuanced picture of how multiple employment conditions cluster together. In that sense, the typological approach provides an opportunity to identify various types of PE and, thereby, expands our understanding of how PE can take expression. The summative score approach provides no insight as to which dimensions or items contribute to the final score. The scoring approach, however, provides a continuum of precariousness, which is an advantage when comparing and identifying degrees of PE. As employment types are not ordinal, they cannot be easily graded or ranked. The typological approach, on the other hand, gives an idea of the size of the workforce that can be considered precariously employed, which is not straightforward in the summative scale approach unless a cut-off score representing PE is decided upon. Another strength of the scoring approach, however, is that it is easily applicable and comparable across time, whereas the number and interpretation of emerging employment types could change among years. Hence, latent class approaches for longitudinal or repeated measures data could instead be applied if multiple years are to be analyzed (39).

A strength of this study is the use of register data, which, unlike survey data, provide objective measures of employment conditions across the entire Swedish work force (including self-employed workers). Further, register data provides opportunities to explore operationalizations of PE across time, sociodemographic and occupational correlates, and a range of register-based outcomes. There are, however, limitations to this study.

There is no formal validation of the typology or summative scale included. However, comparison of scores with employment types and vice versa, supports internal consistency. The sociodemographic and occupational characteristics, as well as the proportions of the precarious employment types falling within the lowest quartile of the summative scale score, supports that both approaches are identifying a similar population.

The register data used did not take informal workers into account. Reports, however, show that approximately 3% of workers in Nordic countries were informal in 2009, which is among the lowest worldwide (34). Further, not all aspects of PE could be optimally operationalized due to the lack of available data. Our items of CBA coverage was based on the probability of receiving occupational pension from the employer, which probably overestimates CBA coverage slightly. Finally, the temporal resolution of one year introduces risk of misclassification in the temporariness and multiple job holding items. Despite these limitations, our study suggests that future research on PE should consider innovative uses of register data.

### Generalizability

Our approach to operationalizing PE could be applied in countries with similar register structure. As political and macroeconomic changes affect the labor market, the emerging typology and score distribution could vary across years. Therefore, studies examining trends over time are needed. We consider both approaches to operationalizing PE to have substantial potential for uses in exposure assessment within epidemiological studies applying register-based outcome measures.

### Concluding remarks

Using the typological approach to operationalize PE identified three types of precarious employment. These provided insight into how precariousness can take expression – in direct employment, solo self-employment or in combined employment (multiple job holding). The scoring approach pointed towards a wide continuum of precarious conditions on the labor market. Gender, age, education and foreign-born status were associated with PE. This was especially notable across age and gender, suggesting that stratified analyses may be appropriate in future studies. Both approaches are promising in terms of exposure assessment: the typological approach being most useful when the experience of different combinations of employment conditions is important; and the summative score approach being most useful when the degree of precariousness is of importance. Etiological studies and research assessing trends over time are needed in order to validate these operationalizations. Register-based operationalizations in countries with similar register structure are encouraged in order to increase international comparability.

## Supplementary material

Supplementary material
