# Supplementary material for: Exploring multidimensional operationalizations of precarious employment in Swedish register data – a typological approach and a summative score approach
Source: Scand J Work Environ Health. 2021 Mar 1;47(2):117–26. doi: 10.5271/sjweh.3928 (PMC8114571; doi:10.5271/sjweh.3928)
Supplement: Supplementary material [file SJWEH-47-117-S001.pdf]

Exploring multi-dimensional operationalizations of precarious employment in Swedish register data – a typological approach and a summative score approach [1]

by Johanna Jonsson, MSc,[2] Nuria Matilla-Santander, PhD, Bertina Kreshpaj, MSc, Cecilia Orellana, PhD, Gun Johansson, PhD, Bo Burström, PhD, Magnus Alderling, MSc, Trevor Peckham, PhD, Katarina Kjellberg, PhD, Jenny Selander, PhD, Per-Olof Östergren, PhD, Theo Bodin, PhD

1. Supplementary material
2. Correspondence to: Johanna Jonsson, Unit of *Occupational* Medicine, Institute of Environmental Medicine, Karolinska Institutet, Solnavägen 4, 11365 Stockholm, Sweden. [E-mail: johanna.jonsson@ki.se]

Figure S1. Flow chart of study population

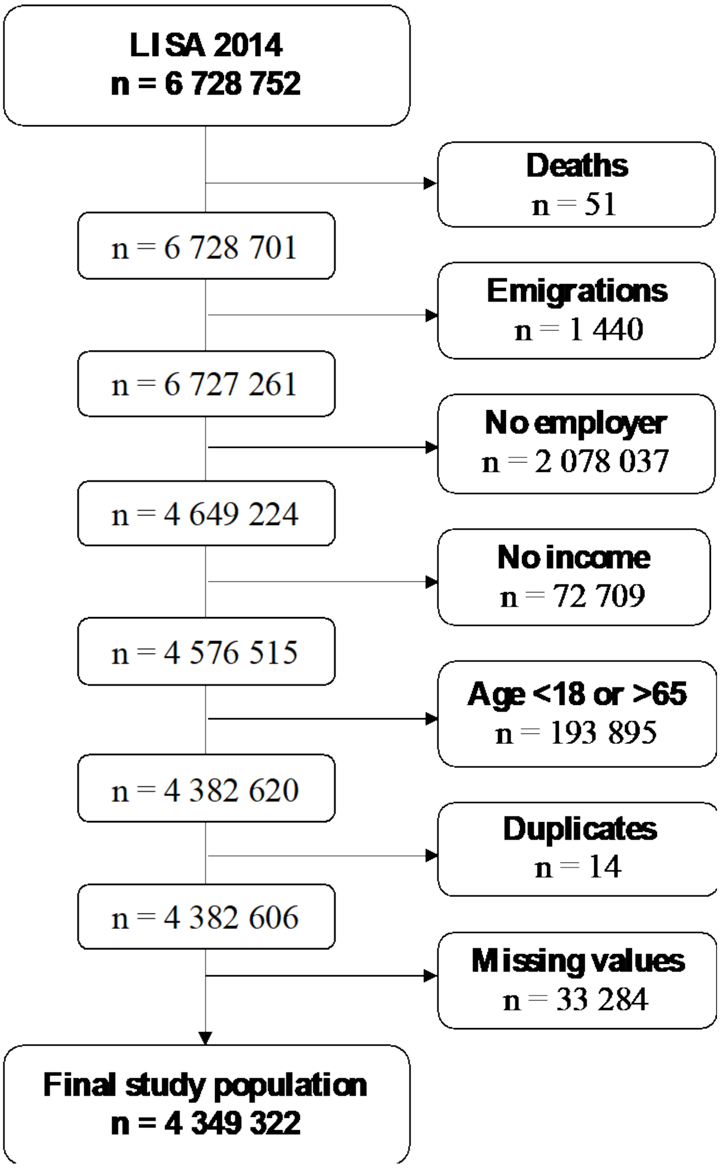

Table S1. Probability of being covered by occupational pension in 2014, stratified by gender		
	Male	Female
Public sector company		
Working in a public sector company	100*	100*
Working in a private sector company – employers branch of economic activity		
Agriculture, forestry, fishing	81.2	79.0
Mining and quarrying; Manufacturing	97.9	97.7
Electricity, gas and air conditioning supply; Water supply; sewerage, waste management and remediation activities	97.8	98.5
Construction	93.9	91.2
Wholesale and retail trade, repair of motor vehicles and motorcycles	94.0	94.7
Transportation and storage	93.4	96.2
Accommodation and food service activities	76.8	86.8
Information, communication	93.9	94.8
Financial and insurance activities	96.6	97.7
Real estate activities	88.7	89.9
Professional, scientific and technical activities; Administrative and support service activities	93.4	93.5
Public administration and defence; compulsory social security	100*	100*
Education	87.1	93.5
Human health and social work activities	95.8	97.4
Arts, entertainment and recreation; Other service activities	83.9	82.4
Working in a private sector company - number of employees in the company		
1	0*	0*
2-5	72.1	70.4
6-10	88.2	87.0
11-50	96.2	95.8
51-100	98.6	98.1
≥101	98.5	98.7
*Imputed values for the sake of the operationlisation		

Table S2. Model fit measures from exploratory and confirmatory latent class analyses				
Exploratory (half sample)				
Cluster solution	AIC	BIC	BIC, adjusted	Entropy
Four clusters	17140689.58	17141382.16	17141207.36	0.85
Five clusters	17092929.43	17093798.30	17093579.02	0.87
Six clusters	17066101.59	17067146.76	17066882.98	0.81
Seven clusters	17050299.87	17051451.33	17051143.06	0.76
Confirmatory, final solution (full sample)				
	AIC	BIC	BIC, adjusted	Entropy
Six clusters	34129266.853	34130369.55	34130105.77	0.81

Table S3. Conditional item probabilities for the final employment typology. SER=Standard Employment Relationship; BO=Business Owners; PER=Precarious Employment Relationship; P-SE=Precarious Self-Employment; P-MJH=Precarious Multiple Job Holders.						
	SER	BO	Proficians	PER	P-SE	P-MJH
Total	60	2	10	22	5	2
Contractual relationship insecurity						
Directly employed by the employer	93	0	78	91	20	11
Employed by an agency	1	0	1	4	0	0
Combination of self-employment and direct employment	7	4	21	5	4	84
Self-employed	0	96	0	0	17	3
Solo self-employed	0	0	0	0	59	2
Contractual temporariness						
Stable employment	92	92	51	8	77	46
Unstable employment	8	8	49	92	24	54
Multiple jobs/economic sectors						
1 job	94	94	11	45	96	4
≥2 jobs	0	4	40	19	3	31
≥2 jobs in >1 economic sector	6	2	49	36	1	65
Income level						
≥200% of the median	6	8	11	1	1	4
120-199% of the median	29	52	44	6	16	24
80-119% of the median	49	31	40	26	26	28
60-79% of the median	11	6	5	20	18	15
<60% of the median	5	4	0	47	39	29
CBA coverage						
>90%	87	20	83	64	0	15
70-90%	11	41	14	24	0	15
≤70%	2	39	3	12	100	70

Table S4. Rationale for labels of the emerging employment typology	
Employment type	Rationale for label
Standard Employment Relationship	Rresembles the SER described in literature, characterized by socially protected, stable, and full-time employment [1].
Business Owners	Reflects self-employed that, in addition to having had the ability to employee employees and as such have better protection against economic downturns, have better conditions compared to solo self-employed.
Proficians	"Proficians", as described by Standing [2], refers to a group of highly skilled and flexible professionals. Previous studies have labelled similar employment types as "portfolio" jobs [3 4].
Precarious Employment Relationship	Embodies the expected features of traditional PE-conditions, including employment instability and poor income [5 6].
Precarious Self-employment	Reflects the precarious and vulnerable situation of solo self-employed on the labor market, having more income- and job-insecurity compared to employed [7] and being more sensitive to economic pressures and downturns than self-employed with employees due to the lack of protection from a larger organization [8].
Precarious Multiple Job holders	As multiple job holding has been argued to be a feature of PE [5 6 9], taken together with poor CBA coverage and poor-moderate income, this employment type was labelled to reflect precarious multiple job holders.

1 Bosch G. Towards a new standard employment relationship in Western Europe. British journal of industrial relations. 2004;42 (4):617-36.

2 Standing G. The precariat: The new dangerous class: Bloomsbury Publishing; 2011.

3 Van Aerden K, Moors G, Levecque K, Vanroelen C. Measuring Employment Arrangements in the European Labour Force: A Typological Approach. Social Indicators Research. 2014;116 (3):771-91. doi: 10.1007/s11205-013-0312-0. PubMed PMID: WOS:000334177600007.

4 Peckham T, Fujishiro K, Hajat A, Flaherty BP, Seixas N. Evaluating Employment Quality as a Determinant of Health in a Changing Labor Market. RSF: The Russell Sage Foundation Journal of the Social Sciences. 2019;5 (4):258-81. doi: 10.7758/rsf.2019.5.4.09.

5 Bodin T, Çağlayan Ç, Garde AH, Gnesi M, Jonsson J, Kiran S, et al. Precarious employment in occupational health - an OMEGA-NET working group position paper. Scandinavian journal of work, environment & health. 2020;46 (3):321-9. doi: 10.5271/sjweh.3860.

6 Kreshpaj B, Orellana C, Burström B, Davis L, Hemmingsson T, Johansson G, et al. What is precarious employment? A systematic review of definitions and operationalizations from quantitative and qualitative studies. Scandinavian journal of work, environment & health. 2020;46 (3):235-47. doi: 10.5271/sjweh.3875.

7 Rasmussen S, Nätti J, Larsen TP, Ilsøe A, Garde AH. Nonstandard Employment in the Nordics–Toward Precarious Work? Nordic Journal of Working Life Studies. 2019;9(S6). doi: https://doi.org/10.18291/njwls.v9iS6.114689.

8 Noack AM, Vosko LF. Precarious jobs in Ontario: Mapping dimensions of labor market insecurity by workers' social location and context. Toronto: Law Commission of Ontario: 2011.

9 Koranyi I, Jonsson J, Rönnblad T, Stockfelt L, Bodin T. Authors' response to" Multiple-job holding is not a type of precarious employment". Scandinavian journal of work, environment & health. 2018;45 (1):100.
